# Education and knowledge helps preventing malaria, but not "degedege"

**DOI:** 10.1186/1475-2875-9-S2-P48

**Published:** 2010-10-20

**Authors:** Astrid Spjeldnæs, Bjørn Blomberg

**Affiliations:** 1 Institute of Medicine, Faculty of Medicine and Dentistry, University of Bergen, Norway; 2 Department of Medicine, Haukeland University Hospital, Bergen, Norway

## Background

Socio-cultural aspects and the gap between the local population and the policy-makers present barriers to malaria-control programmes [1]. Thus, it is essential for malaria prevention efforts that policy-makers acknowledge the local perceptions and beliefs in the rural areas affected by malaria.

## Methods

This study assesses knowledge, attitudes and behaviour towards malaria and malaria-like illnesses. A cross-sectional survey was conducted in Rufiji, Tanzania. A scoring system was used to capture the participants' knowledge of malaria (KoM) and another for the preventive actions of malaria (AoM). Logistic regression was used to assess factors associated with high KoM and AoM.

## Results and conclusion

Most of the participants had knowledge of the main symptoms of malaria, its cause and the use of nets as prevention, but fewer actually put their knowledge into action. Education and age 30 to 49 were significantly associated with higher knowledge score (OR: 2.81 (1.28 - 6.19) and 3.05 (1.25 - 7.43) respectively). Education was associated with higher score in preventive action (OR: 7.12 (2.20 - 23.07)). Having middle or higher levels of education was not significantly associated with higher knowledge and action score, probably because few participants belonged to this group. The study suggested that the population had good knowledge of mild to moderate malaria. However, the study population also had a conception of a condition called "degedege", which features fever and convulsions. The traditional concept of "degedege" may encompass a number of serious conditions in western medical terminology, including cerebral malaria, bacterial meningitis and epilepsy. Most participants considered "degedege" to have supernatural causes, and that it is not preventable by net-use, and that it should be treated by a local healer. These beliefs can be a reason for delayed care-seeking. This may also partially explain why malaria is not perceived as a serious threat in the area, and thus could contribute to the lack of preventive actions.
